# Study on the physicochemical and flavor characteristics of air frying and deep frying shrimp (crayfish) meat

**DOI:** 10.3389/fnut.2022.1022590

**Published:** 2022-12-01

**Authors:** Mingzhu Zhou, Gangpeng Shi, Yi Deng, Chao Wang, Yu Qiao, Guangquan Xiong, Lan Wang, Wenjin Wu, Liu Shi, Anzi Ding

**Affiliations:** ^1^Key Laboratory of Cold Chain Logistics Technology for Agro-Product, Ministry of Agriculture and Rural Affairs, Institute of Agro-Product Processing and Nuclear Agricultural Technology, Hubei Academy of Agricultural Sciences, Wuhan, China; ^2^Key Laboratory of Fermentation Engineering (Ministry of Education), Hubei Key Laboratory of Industrial Microbiology, Hubei Provincial Cooperative Innovation Center of Industrial Fermentation, Hubei University of Technology, Wuhan, China

**Keywords:** shrimps, air frying, deep frying, physicochemical properties, flavor characteristics

## Abstract

This study aimed to compare the changes in the quality characteristics of air-fried (AF) shrimp meat and deep-fried (DF) shrimp meat at different frying temperatures (160, 170, 180, 190°C). Results showed that compared with DF, the moisture and fat content of air-fried shrimp meat (AFSM) was lower, while the protein content was higher. At the same frying temperature, the fat content of the AFSM was 4.26–6.58 g/100 g lower than that of the deep-fried shrimp meat (DFSM). The smell of the AFSM and DFSM was significantly different from that of the control group. The results of the electronic tongue showed that each of the two frying methods had its flavor profile. Gas chromatography-ion mobility spectrometry (GC-IMS) identified 48 compounds, and the content of volatile compounds detected in AFSM was lower than that in DFSM. Among them, the highest level of volatile compound content was found in the DF-190. E-2-pentenal, 2-heptenal (E), and methyl 2-methyl butanoate were identified only in DFSM. In addition, a total of 16 free amino acids (FAAs) were detected in shrimp meat. As judged by sensory evaluation, the AFSM at 170°C was the most popular among consumers.

## Introduction

Deep-fried (DF) is a traditional method of cooking with a century-old history (1). The frying process concerns the transfer of heat between the food and the hot oils. Frying could produce some unique flavor characteristics, owing to chemical reactions like browning reaction, Millard reaction, caramelization, and lipid oxidation, which are attractive to consumers (2). In addition, some free amino acids (FAAs) produced by proteolysis and Strecker degradation may be liable for the distinctive flavor of deep-fried shrimp meat (DFSM). But unacceptable characteristics to consumers also originated during DF. The high oil of fried food was getting more and more attention from consumers. Research has determined that excessive intake of fats enhances the hazard of hypertension and obesity (3). Also, the traditional frying method needs high energy. Therefore, it is necessary to develop a technology to reduce the oil content of fried products without compromising product quality. So far, modified methods of alternative frying technology have been developed, including vacuum frying, AF, microwave frying, microwave-assisted vacuum frying, and ultrasound-treated frying (4).

Air-fried (AF) is a rapid cooking technology. This is achieved by spraying hot air around the ingredients to promote uniform contact between the food and the hot air. This process is carried out in an air fryer apparatus that simulates the movement of heat flow in boiling oil, dehydrating the sample and making it crispy. Compared to traditional DF, AF can lessen the oil content of fried foods. Also, it can form the characteristic shell of fried food due to dehydration (5). Moreover, AF has the advantage of inhibiting the formation of acrylamide (6). Compared with DF foods, the oil content of AF food exhibits 70–80% lower and the acrylamide content can be reduced by about 90% (7). The main component of French fries is starch, but crawfish shrimp meat contains a lot of protein as well as a small amount of fat, which results in a significantly different frying mechanism between the two kinds of food. Cao et al. (8) found that the oil content of AF chicken nuggets was 25% lower than that of DF chicken nuggets. AF was a healthier frying method that can reduce oxidative deterioration of lipids.

Procambarus clarkii is a crustacean. It contains vitamins, polyunsaturated fatty acids (PUFA), and protein (9). Diverse cooking techniques are acting on shrimp meat, comprising frying, baking, steaming, etc. (10). Among them, frying is popular with people due to its appearance, texture, taste, and flavor. However, the health of fried foods with high oil content has attracted increasing attention recently, and researchers have revealed that excessive intake of fat will increase the risk of obesity, high blood pressure, and cardiovascular disease (3). In recent years, AF has been applied to the processing technology of fish and shrimp. Joshy et al. (11) have used the AF technique to prepare a fish cutlet-a popular fish snack with low-fat content and better protein content. Yu et al. (12) found that AF can be regarded as a healthier technique than deep-fat-fried surimi. Heat treatment has been shown to induce lipid oxidation due to the disruption of cell membranes and hemoglobin denaturation, as well as the release of free iron, which promotes lipid oxidation of the product (13). This in turn causes losses to the fried product, and the extent of losses depends on cooking conditions, etc. (14). Therefore, conditions such as cooking temperature have an important effect on the lipid oxidation reaction of crayfish meat (14). And the result of air-frying on the lipid oxidation in prawns with different temperatures was evaluated by Song et al. (9). The results show that temperature plays an important role in influencing the lipid profile of air-fried shrimp and low-temperature turn frying (140–160°C) is less detrimental to the quality of shrimp meat. To date, there has been no comparative study on the physicochemical and flavor characteristics of air-fried shrimp meat (AFSM) and DFSM.

This study would compare the effect of AF and DF on the physicochemical and flavor characteristics of shrimp meat, and discuss the different operating temperatures of frying on the quality characteristics of shrimp meat, namely, the nutrients (moisture, oil, and protein content), physicochemical (color, texture, sensory), and flavor. Meanwhile, it can also identify the optimal AF and DF temperature for producing shrimp meat. These studies provide a basis for the development of healthy fried food.

## Materials and methods

### Raw materials

Fresh crayfish was obtained from a local market in Wuhan (Longitude: 114.29, Latitude: 30.48), Hubei province of China, and immediately transported to the laboratory at 0–4°C to maintain vigorous vitality. Crayfish with similar body weights (30.00 ± 2.00 g) were selected as experimental samples. The shrimp meat of the crayfish was taken out and washed with pure water, then the surface moisture was blot-dried using absorbent papers.

### Frying process

The shrimp meat was divided into three groups, each weighing 100.00 ± 2.00 g. The non-fried shrimp meat was used as the control group. The other two groups were DF and air fried, respectively. For DF, a household deep fryer (model: XJ-15301, Shenzhen, China) with a rated power of 1400 W was used. The shrimp meat was fried in 2 L soybean oil at 160, 170, 180, and 190°C, respectively. Considering that the DF time should not be too long (15), the frying time was set to 2 min. Because AF is an oil-free food processing technology, no oil was applied during the frying of the shrimp meat process. For AF, an air fryer (model: S-2021TS, Shenzhen, China) with a rated power of 1300 W was used, which was preheated at the set temperature for 3 min before the experiment. The shrimp meat was fried in an AF pan at 160, 170, 180, and 190°C for 10 min, and flipped every 5 min. Subsequently, shrimp meat was removed from fryers and cooled down to room temperature before further analysis.

### Nutritional composition analysis

Proximate analysis of DF, AF, and control samples was carried out as per AOAC (16). Moisture, protein, fat, and ash contents of shrimp meat at varying process conditions were measured.

### Color and texture analysis

The color meter (model: CR-400, Konica Minolta Holdings, Inc. (Tokyo, Japan) was used to measure the surface color of shrimp meat. The color of the shrimp meat surface was expressed as L* (lightness), a* (redness-greenness), and b* (yellowness-blueness).

Texture profile analysis was performed according to Fan et al. (17). The shrimp meat as described in Section “Frying process” were placed on the loading platform of a TA-XT 2i/50 texture analyzer (Stable Micro Systems, Ltd., Surrey, UK). A P/2 cylindrical probe was used to measure the surface hardness and springiness of the shrimp meat.

### Electronic nose analysis

The smell analysis was performed using a Pen III (Airsense, Germany) E-nose system. The PEN III system contained 10 different MOS (metal oxide semiconductor) and the sensors are described in Table 1. Approximately 2.00 g of the shrimp meat and 2 mL 0.18 g/mL NaCl were placed into a 40 ml headspace vial and incubated at 60°C for 30 min. The headspace gas was extracted into the MOS sensors at a constant rate of 300 mL min^–1^. The flush time and measurement time were 100 and 120 s, respectively.

**TABLE 1:** Effect of frying method and temperature on basic nutritional components, color, and texture of shrimp meat.

Group	Moisture content (g/100 g)	Fat (g/100 g)	Protein (g/100 g)	L*	a*	b*	Surface hardness (N)	Springiness
CK	81.04 ± 0.47^a^	0.50 ± 0.16^h^	16.44 ± 0.12^f^	51.74 ± 0.09^c^	1.72 ± 0.15^de^	11.56 ± 0.11^d^	24.63 ± 5.58^f^	22.92 ± 8.34^d^
DF-160	72.72 ± 0.08^b^	5.57 ± 0.06^d^	19.65 ± 1.36^e^	73.93 ± 0.25^b^	1.07 ± 0.16^e^	11.89 ± 0.81^d^	83.22 ± 9.22^e^	52.63 ± 1.83^ab^
DF-170	69.91 ± 0.42^c^	6.19 ± 0.14^c^	21.12 ± 0.11^d^	74.49 ± 1.36^b^	2.76 ± 0.35^cd^	16.41 ± 1.80^c^	95.77 ± 3.37^de^	53.09 ± 0.79^a^
DF-180	67.16 ± 0.10^de^	7.70 ± 0.28^b^	22.97 ± 0.16^c^	74.90 ± 2.23^b^	3.43 ± 0.25^abc^	17.84 ± 1.06^bc^	111.55 ± 11.13^de^	51.39 ± 2.47^ab^
DF-190	64.70 ± 0.11^f^	9.24 ± 0.09^a^	24.01 ± 0.03^c^	72.50 ± 2.82^b^	3.90 ± 0.74^ab^	18.49 ± 0.29^bc^	134.76 ± 15.70^d^	49.35 ± 1.44^abc^
AF-160	68.06 ± 0.16^d^	1.31 ± 0.09^g^	28.37 ± 0.21^b^	77.36 ± 0.51^a^	1.68 ± 0.38^de^	18.13 ± 3.02^bc^	188.18 ± 19.56^c^	48.18 ± 1.38^bc^
AF-170	66.78 ± 0.44^e^	1.48 ± 0.15^fg^	29.13 ± 0.13^b^	74.76 ± 0.55^b^	2.73 ± 0.40^cd^	18.47 ± 1.65^bc^	198.33 ± 20.18^c^	51.07 ± 0.70^ab^
AF-180	63.40 ± 1.03^g^	1.75 ± 0.06^f^	31.81 ± 0.09^a^	74.68 ± 0.43^b^	3.14 ± 0.69^bc^	19.39 ± 1.20^b^	242.00 ± 57.45^b^	48.83 ± 1.00^abc^
AF-190	62.35 ± 0.16^h^	2.66 ± 0.31^e^	32.50 ± 0.23^a^	72.59 ± 0.66^b^	4.34 ± 1.27^a^	22.82 ± 0.71^a^	353.41 ± 65.65^a^	46.31 ± 1.20^c^

Values carrying different letters at the same time indicate statistically significant differences according to Duncan’s multiple range test (*p* < 0.05). DF, shrimp meat was treated with deep frying; AF, shrimp meat was treated with air frying; 160, 170, 180, 190: The fried temperature of shrimp meat is expressed as 160, 170, 180, and 190°C.

### Electronic tongue analysis

According to Miao et al. (18), shrimp meat was detected by using ASTREE II (Alpha M.O.S France). Approximately 2.00 g of shrimp meat was mixed with 100 ml of distilled water to extract taste substances. The mixture was kept for 30 min and centrifuged, then the water phase was obtained as a measurement sample. Sensors were calibrated and diagnosed before the shrimp meat test. Subsequently, Sensors were dipped in the extract solution.

### Gas chromatography-ion mobility spectrometry

According to Xing et al. (19), an analysis of shrimp meat was performed using a GC-IMS instrument (FlavourSpec^®^ in the G.A.S. Department of Shandong HaiNeng Science Instrument Co., Ltd., Shandong, China). Shrimp meat (1.00 g) was homogenized with 10 ml distilled water and 1.00 g NaCl and incubated in a 20 ml headspace bottle for 15 min at 80°C. Gas chromatography (GC) was performed with a column (FS-SE-54-CB-1, 15 m × 0.53 mm × 1.0 μm) to separate the volatile components. The chromatographic was programmed as follows: 2 ml/min for 2 min, 10 ml/min for 8 min, 100 ml/min for 10 min, and 150 ml/min for 10 min. Nitrogen at a flow rate of 150 ml/min was used as the drift gas for IMS, and the temperature was set at 45°C. The GC-IMS data analysis used the functional software Laboratory Analytical Viewer and two plug-ins Reporter and Gallery Plot to construct a 2D topography and fingerprint of the volatile compounds of the samples, and the GC-IMS from the instrument software National Institute of Standards and Technology (NIST) and G.A.S. The volatile compounds were identified by the drift time (DT) of the standard compounds in the database. The relative amounts of the identified volatiles were expressed as the average of the peak areas. Peak intensities of volatile compounds detected by GC-IMS were used for principal component analysis (PCA) to distinguish the differences between different frying temperatures.

### Free amino acid determination

Free amino acids were measured according to Yu et al. (20). Amino Acid Automatic Analyzer L-8900 (Hitachi, Tokyo) was employed to analyze shrimp meat. Shrimp meat (2.00 g) was homogenized with 15 ml of 5% trichloroacetic acid for 1 min. Then it was placed in a refrigerator at 4°C for 2 h and centrifuged at 9,000 r/min for 15 min with 10 ml supernatant. The 5 ml supernatant was taken out, and the pH was adjusted to 2.0 with 6 mol/L NaOH, then the volume was expanded with distilled water to 10 ml. One milliliter of the extract was filtered using a 0.22 μm membrane filter and applied to an automatic amino acid analyzer.

### Sensory evaluation

The sensory evaluation group consisted of 5 boys and 5 girls with an age range of 23–28 years. These panelists received 8 weeks of training, twice a week for 30 min each time. Samples were then randomly assigned to 10 trained panelists. Each panelist scored the samples for each treatment. The basis of sensory scoring was developed by Cao et al. (8) which describes each classification in a 1–5 score. Each indicator is described in a graded manner and divided into three levels: ideal (5 points), more ideal (4 points), moderate (3 points), poor (2 points), and unsatisfactory (1 point). The sensory characteristics of DFSM and AFSM were evaluated by four sensory indicators including color, odor, taste, and texture.

### Statistical analysis

Statistical analysis was performed using Origin 2017 (Microcal Software, Inc., Northampton, MA, USA) and DPS software (Zhejiang University, Hangzhou, China). All the data were obtained from at least three repeats. Differences among samples were evaluated using analysis of variance (ANOVA) and Duncan’s multiple-range test (*P* < 0.05).

## Results and discussion

### Changes in basic nutritional components, color, and texture

The nutritional ingredients, chromaticity, and texture properties of fried shrimp meat were shown in Table 1. It can be observed that the DFSM and AFSM both decreased in moisture content compared to the control group. The moisture content of the AFSM was slightly lower than that of the DFSM. The reason might be that the AFSM was prepared in a closed system, which would transfer water molecules through a rapid air-flowing mechanism. Meanwhile, the moisture content of AFSM and DFSM reduced with the increase in frying temperature. It may be that the transfer rate of water molecules was accelerated with the increase of AF temperature; while with the increase of DF temperature, the exogenous oils were transferred to the shrimp meat tissues, then the water was accelerated to evaporate. On the other hand, the fat content of raw shrimp meat was 0.50 g/100 g, while that of cooked shrimp meat varied between 5.57 g/100 g and 9.24 g/100 g in DF and between 1.31 and 2.66 g/100 g in AF. On average, the fat content of the AFSM was 74% lower than that of the DFSM. More specifically, the fat content of AF-160, AF-190, DF-160, and DF-190 was 1.31, 2.66, 5.57, and 9.24 g/100 g, respectively. The AFSM was reduced in fat content in contrast to the DFSM because of the absence of oil. This result was following studies conducted on AF fish (12). According to the study of Moreira and Barrufet (21), the high-fat content of DFSM may be stemmed from the equilibrium reaction between the adhesion and drainage of the shrimp surface when the shrimp meat is removed from the oil. Furthermore, when the frying temperature increased, the fat content of samples was also found to be increasing. Since no oil was used for frying, the enhancement of fat content in AFSM may be due to the expulsion of fat from shrimp meat as no other liquid would replace the water removed from the pores of the shrimp meat; With the increase in temperature, the moisture of fried shrimp meat was accelerated to evaporate, and the content of exogenous oil into the shrimp meat tissue increased, which made the oil content increased. In addition, the protein content of raw shrimp meat was 16.44 g/100g, and the AFSM was increased in protein content compared to the DFSM. The protein content increased significantly at increasing frying temperature. This is mainly due to the variation in moisture content. In summary, it shows that AFSM possesses the characteristics of low fat and high protein.

Color parameter is one of the most important quality attributes of fried foods, and also plays an important role in appearance evaluation, which directly impacts consumers’ liking for fried foods. The color change of fried food is mainly related to non-enzymatic browning reactions such as chemical oxidation, caramelization, and Maillard reaction (22). The variation of L* (lightness), a* (redness-greenness), and b* (yellowness-blueness) in AFSM and DFSM was shown in Table 1. The L* value is a key parameter for the brightness of fried products (23). The L* values of AF and fried shrimp were significantly higher compared to the control group (51.74), which indicated that the lightness of AF and fried shrimp was higher than that of raw shrimp meat. As the AF temperature increased, the lightness of shrimp meat gradually decreased from 77.36 to 72.59, which was attributed to the water loss and reduced light reflection, thus leading to the browning and darkening of the surface of AFSM (14); However, the redness and yellowness value of shrimp meat gradually increased with the increase of the frying temperature. The a* and b* value of the control group was 1.72 and 11.56, and they rose to 3.90 and 18.49 after DF under 190°C. Conclusively, the values of redness and yellowness were the highest in AFSM. It was believed that a high redness value was directly associated with the generation of acrylamide (24). In general, a high yellowness value suggested more golden shrimp meat, which was acceptable and desirable for fried products.

The texture changes of fried products might depend on the joint action of heat and mass transfer and chemical reactions occurred in the frying process (25). The textural changes are mainly due to protein denaturalization, water evaporation, and tissue browning (26). These changes would greatly affect the likability of the consumers on the products. As presented in Table 2, the surface hardness and springiness of the control group were 24.63 N and 22.92 N. The surface hardness of frying shrimp meat increased as the frying temperature rose, which reached the maximum value of 353.41 N and 134.76 N at 190°C for AFSM and DFSM, respectively. The surface hardness of AFSM was significantly higher than DFSM (*p* < 0.05), and the reason might be that the surface layer of the shrimp meat gradually forms a hard shell during the air-frying process (8). There was no significant difference in springiness between AFSM and DFSM, but a sudden drop of springiness occurred when the temperature climbed up to 190°C; Such variation in springiness may be caused by the uniform and uneven thermal denaturation of myosin and actin (27).

**TABLE 2:** The performance description of the sensor.

Array serial number	Sensor name	Representative material species	Performance description
1	W1C	Aromatic	Aromatic constituents, benzene
2	W5S	Broad range	High sensitivity and sensitive to nitrogen oxides
3	W3C	Aromatic	Sensitive aroma, ammonia
4	W6S	Hydrogen	Mainly selective for hydrides
5	W5C	Arom-aliph	Short-chain alkane aromatic component
6	W1S	Broad-methane	Sensitive to methyl
7	W1W	Sulfur-organic	Sensitive to sulfides
8	W2S	Broad-alcohol	Sensitive to alcohols, aldehydes, and ketones
9	W2W	Sulph-chlor	Aromatic ingredients, sensitive to organic sulfides
10	W3S	Methane-aliph	Sensitive to long-chain alkanes

### Electronic nose response

**FIGURE 1:**
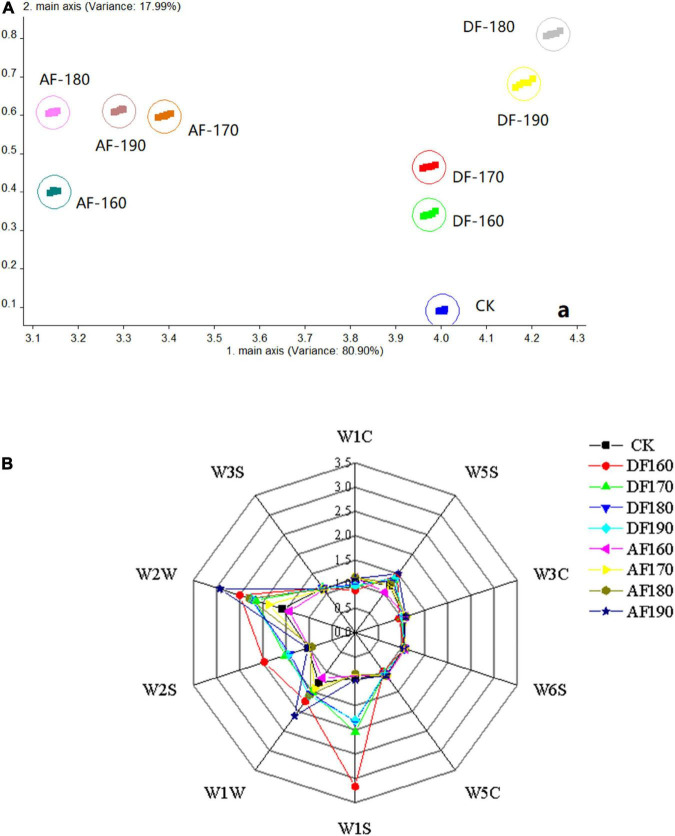
Effect of frying method and temperature on the principal component analysis (PCA) of shrimp meat smell **(A)** and sensor signal value **(B)**. DF, shrimp meat was treated with deep frying; AF, shrimp meat was treated with air frying; 160, 170, 180, 190: The fried temperature of shrimp meat is expressed as 160, 170, 180, and 190°C.

Electronic noses can be used to determine the odor of a sample, and slight variations in volatile compounds in the sample may lead to differences in sensor response. Data were collected through sample testing and the results of 118–120 s were selected for principal component analysis, which was used to reflect the differences between samples. As shown in Figure 1A, it indicates that the first and second principal components were 80.90 and 17.99%, respectively. This principal component accounted for 98.89% of the total variance. In the scatter plot, shrimp meat from different treatments was well-separated. The AF group and DF group were respectively located on the left and right sides of the PCA, indicating that the smell of the AFSM and DFSM was significantly different from that of the CK group. In AF, the smell of shrimp meat fried at 160°C was significantly different from that fried at 170, 180, and 190°C. In DF, the smell attributes of shrimp meat fried at 160 and 170°C were similar, and there was no difference between shrimp meat fried at 180 and 190°C. E-nose showed a good ability to differentiate fried shrimp through 10 sensors. Response values from 10 sensors were shown in Figure 1B. The sensors W2W, W2S, W1W, and W1S contributed most to the fried shrimp meat, suggesting that sulph-chlor, broad-alcohol, sulfur-organic, and broad-methane had the greatest influence on the odor of fried shrimp (Table 2). Meanwhile, Figure 1B also indicated that W2W, W2S, W1W, and W1S sensors could be effective to distinguish CK, DF, and AF samples. The flavor detected by W2W, W2S, and W1S likely made the greatest contribution to the odor contour of the DFSM, which was probably owing to the high degree of lipid oxidation during deep-frying (28). The lipid oxidation in the frying oil could directly affect the flavor of DF food (29).

### Electronic tongue

**FIGURE 2:**
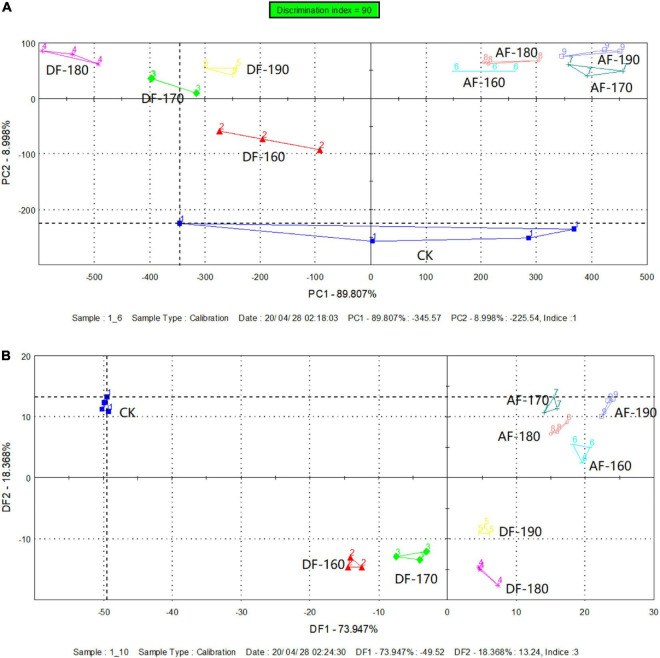
Effect of frying method and temperature on principal component analysis (PCA) **(A)** and discriminant factor analysis (DFA) **(B)** of shrimp meat taste. DF, shrimp meat was treated with deep frying; AF, shrimp meat was treated with air frying; 160, 170, 180, 190: The fried temperature of shrimp meat is expressed as 160, 170, 180, and 190°C.

The electronic tongue is a simulation of the human tongue to analyze, identify and judge the sample measured, and the data obtained is processed by multivariate statistical methods to quickly reflect the quality information of the sample as a whole and achieve the identification and classification of the sample. Figure 2 exhibited the principal component analysis (PCA) and discriminant factor analysis (DFA) diagram which summarized the overall connections between the AFSM and the DFSM. For shrimp meat, the PC1 and PC2 explained 98.81% of the total variation, and the PC1 and PC2 were, respectively, 89.81 and 9.00% (Figure 2A), indicating that the PC1 and PC2 cover the vast majority of taste information of the shrimp meat (30). Meanwhile, the discrimination index reached 90, which certificated that there was a dramatic difference between the tastes of shrimp meat of different frying methods. Compared with the control group, significant changes from the DF and AF groups proved that the taste of shrimp meat depended on the frying method. This difference might be due to the lipid oxidation and Maillard reactions (28). PCA was performed to identify the shrimp meat with different treatments. To better investigate the differences in shrimp meat under different temperatures, DFA was used (Figure 2B). DF1 and DF2 explained 73.95 and 18.37% of the sample variances, respectively. The total contribution (92.32%) indicated that DFA could better distinguish these samples. The data collected by the two frying methods were distributed in different areas of the DFA diagram, indicating that the shrimp meat showed different flavor characteristics after different frying treatments. In addition, there was no overlap in the distribution of data collected at different temperatures for the same frying method, which indicated that the flavor of shrimp was affected by the frying temperature. The result agreed with that obtained by E-nose.

### Gas chromatography-ion mobility spectrometry

**FIGURE 3:**
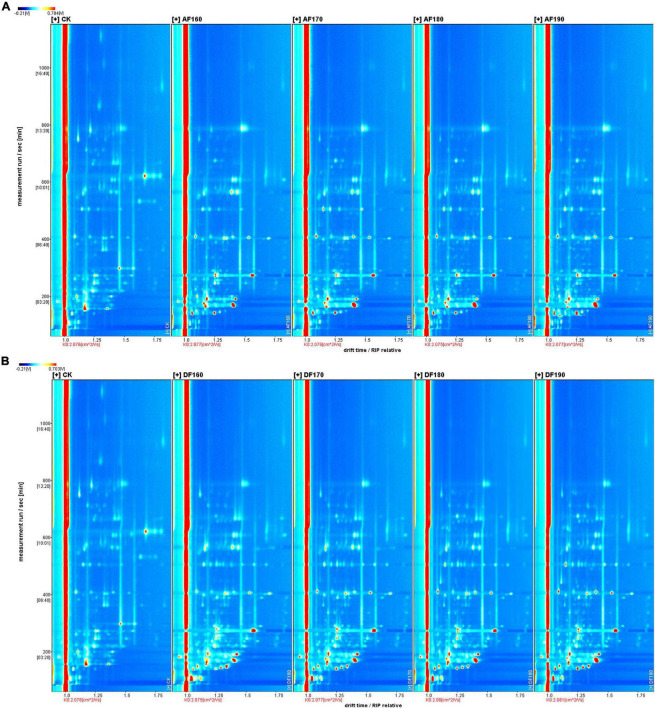
Effect of frying temperature on the gas-phase ion mobility spectrum (GC-IMS) of AFSM **(A)** and the GC-IMS of DFSM **(B)**. DF, shrimp meat was treated with deep frying; AF, shrimp meat was treated with air frying; 160, 170, 180, 190: The fried temperature of shrimp meat is expressed as 160, 170, 180, and 190°C.

**FIGURE 4:**
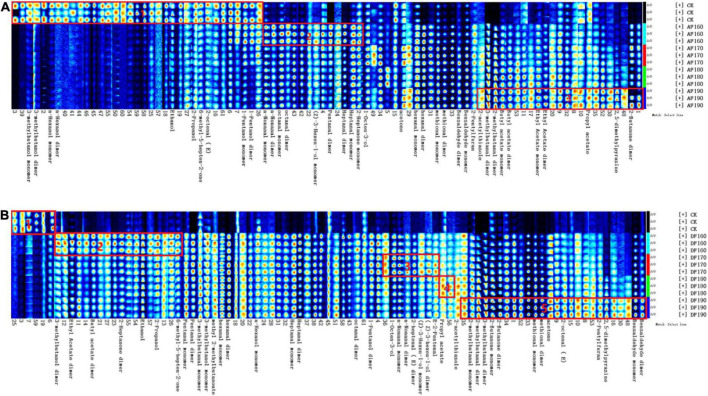
Effect of frying temperature on the volatile flavor compounds of AFSM **(A)** and the volatile flavors of DFSM **(B)**. DF, shrimp meat was treated with deep frying; AF, shrimp meat was treated with air frying; 160, 170, 180, 190: The fried temperature of shrimp meat is expressed as 160, 170, 180, and 190°C.

The differences in volatile compounds in shrimp meat with different frying methods were analyzed by GC–IMS. In Figure 3, the ordinate and the abscissa denote the retention time and drift time, respectively. The red vertical line in the figure represents the reactive ion peak (RIP). Each point on the right of RIP represents a volatile compound. The darker the color, the higher the concentration. To comprehensively compare the differences between volatile compounds, the peak signal graph was listed by the gallery plot plug-in of the LAV software for intuitive comparison. In Figure 4, each row represents all detected signal peaks, and each column represents the signal peak of the same volatile compound under different frying methods and temperatures. The brighter the signal peak, the higher the content of the compound. Due to their different concentrations, it was observed that certain single compounds might produce dimers (31). The formation of dimers was also associated with its high proton affinity, consistent with the research (32). As shown in Figure 4, fingerprinting was used to analyze the differences in the volatile compound content of shrimp meat under different frying methods and temperatures. A total of 48 typical target compounds were identified by the GC × IMS Library (Figure 4 and Tables 3, 4). The signal intensities of 3-methylbutanol, and n-hexanol in fresh shrimp meat were much higher than that in AFSM, while fresh shrimp meat was much lower than DFSM. In addition, E-2-pentenal, 2-heptenal (E), and methyl 2-methylbutanoate were identified only in DFSM.

**TABLE 3:** Types and peak areas of volatile flavor compounds in fried crayfish shrimp meat.

Group	Volatile flavor compounds (Peak volume)						
	
	Aldehydes	Ketones	Alcohols	Ester	Pyrazines	Others	Total
CK	5496.75 ± 821.66^e^	1546.49 ± 58.14^c^	3466.02 ± 394.49^e^	629.29 ± 44.68^g^	100.71 ± 21.34^f^	52.07 ± 15.79^d^	11291.32 ± 1273.54^d^
DF-160	18048.29 ± 286.80^cd^	3322.79 ± 406.39^ab^	8821.25 ± 20.43^a^	3465.77 ± 227.21^a^	138.86 ± 19.76^e^	109.57 ± 10.68^c^	33906.52 ± 765.19^b^
DF-170	20269.01 ± 809.49^bc^	3809.61 ± 210.49^ab^	8021.44 ± 210.53^b^	2512.76 ± 159.17^c^	189.65 ± 25.14^d^	140.54 ± 4.75^b^	34943.00 ± 1245.11^ab^
DF-180	20417.28 ± 799.82^b^	3778.44 ± 358.69^ab^	7456.99 ± 233.03^c^	2309.09 ± 49.43^d^	410.73 ± 22.77^b^	145.45 ± 12.86^b^	34517.98 ± 869.06^b^
DF-190	22201.41 ± 376.47^a^	4277.52 ± 256.85^a^	6676.62 ± 130.39^d^	2791.46 ± 46.86^b^	596.59 ± 14.92^a^	170.52 ± 3.73^a^	36714.12 ± 444.52^a^
AF-160	19123.21 ± 790.03^cd^	3502.92 ± 185.13^ab^	2506.86 ± 28.23^fg^	954.12 ± 22.54^f^	148.12 ± 2.44^e^	114.82 ± 11.59^c^	26350.05 ± 932.18^c^
AF-170	19866.67 ± 437.90^bc^	3956.14 ± 331.90^ab^	2539.78 ± 165.08^f^	1030.52 ± 30.99^ef^	196.38 ± 13.33^d^	119.61 ± 20.72^c^	27709.11 ± 326.03^c^
AF-180	20012.88 ± 554.68^bc^	3074.23 ± 500.60^b^	2175.09 ± 81.31^g^	1102.97 ± 25.63^ef^	244.71 ± 15.56^c^	106.08 ± 5.70^c^	26715.95 ± 992.71^c^
AF-190	20709.24 ± 765.51^b^	3464.52 ± 1217.99^ab^	2269.30 ± 68.82^fg^	1162.58 ± 77.66^e^	410.42 ± 11.61^b^	117.10 ± 12.01^c^	28133.16 ± 1928.08^c^

Values carrying different letters at the same time indicate statistically significant differences according to Duncan’s multiple range test (*p* < 0.05). DF, shrimp meat was treated with deep frying; AF, shrimp meat was treated with air frying; 160, 170, 180, 190: The fried temperature of shrimp meat is expressed as 160, 170, 180, and 190°C.

**TABLE 4:** Qualitative results of GC-IMS of fried crayfish shrimp meat.

Count	Compound	CAS	Formula	MW	RI	RI reference value (44, 45)	Rt [s]	Dt [RIPrel]	Comment
1	2-Butanone	C78933	C_4_H_8_O	72.1	586.0	587.4	141.297	1.06217	Dimer
2	2-Butanone	C78933	C_4_H_8_O	72.1	587.6	604.1	142.116	1.24556	
3	Pentanal	C110623	C_5_H_10_O	86.1	689.9	699.2	192.075	1.18273	
4	Pentanal	C110623	C_5_H_10_O	86.1	689.9	699.2	192.075	1.42469	Monomer
5	Ethyl acetate	C141786	C_4_H_8_O_2_	88.1	601.2	618.1	148.668	1.09698	Dimer
6	Ethyl acetate	C141786	C_4_H_8_O_2_	88.1	603.4	619.6	149.76	1.33725	Monomer
7	2-Methylbutanal	C96173	C_5_H_10_O	86.1	662.1	668.8	178.152	1.15896	Dimer
8	2-Methylbutanal	C96173	C_5_H_10_O	86.1	655.9	667.4	175.149	1.40092	Monomer
9	3-Methylbutanal	C590863	C_5_H_10_O	86.1	636.1	633.8	165.594	1.17254	Dimer
10	3-Methylbutanal	C590863	C_5_H_10_O	86.1	638.4	645.3	166.686	1.40602	Monomer
11	Hexanal	C66251	C_6_H_12_O	100.2	789.7	802.2	275.74	1.25445	Dimer
12	Hexanal	C66251	C_6_H_12_O	100.2	788.3	801.1	274.122	1.56593	Monomer
13	Butyl acetate	C123864	C_6_H_12_O_2_	116.2	803.6	813.4	291.91	1.23439	Dimer
14	Butyl acetate	C123864	C_6_H_12_O_2_	116.2	801.9	815.4	289.889	1.62138	Monomer
15	1-Pentanol	C71410	C_5_H_12_O	88.1	756.5	770.9	246.632	1.24973	Dimer
16	1-Pentanol	C71410	C_5_H_12_O	88.1	757.0	773.9	247.036	1.50811	Monomer
17	3-Methylbutanol	C123513	C_5_H_12_O	88.1	724.4	730.7	220.355	1.23911	Dimer
18	3-Methylbutanol	C123513	C_5_H_12_O	88.1	724.9	730.7	220.759	1.49042	Monomer
19	Methional	C3268493	C_4_H_8_OS	104.2	901.1	907.3	412.445	1.08947	Dimer
20	Methional	C3268493	C_4_H_8_OS	104.2	899.9	909.9	410.052	1.39814	
21	2-Heptanone	C110430	C_7_H_14_O	114.2	887.6	891	389.943	1.26068	
22	2-Heptanone	C110430	C_7_H_14_O	114.2	887.4	896	389.609	1.63488	Monomer
23	Heptanal	C111717	C_7_H_14_O	114.2	897.2	887.8	404.946	1.3282	Dimer
24	Heptanal	C111717	C_7_H_14_O	114.2	896.5	889.3	403.612	1.69897	Monomer
25	n-Non-anal	C124196	C_9_H_18_O	142.2	1104.6	1109.5	789.187	1.47463	Dimer
26	n-Non-anal	C124196	C_9_H_18_O	142.2	1103.6	1104	787.405	1.9464	Monomer
27	Octanal	C124130	C_8_H_16_O	128.2	1003.8	1007.2	609.194	1.39423	Dimer
28	Octanal	C124130	C_8_H_16_O	128.2	1004.1	1007.0	609.788	1.82353	
29	6-Methyl-5-hepten-2-one	C110930	C_8_H_14_O	126.2	986.3	—	576.522	1.17579	
30	Benzaldehyde	C100527	C_7_H_6_O	106.1	949.9	963.7	506.426	1.15303	
31	Benzaldehyde	C100527	C_7_H_6_O	106.1	949.6	962.8	505.832	1.46704	
32	Ethanol	C64175	C_2_H_6_O	46.1	514.8	512.3	106.862	1.04563	
33	Acetone	C67641	C_3_H_6_O	58.1	520.9	519.7	109.795	1.11586	
34	2-Propanol	C67630	C_3_H_8_O	60.1	520.1	514.0	109.429	1.08309	
35	Propyl acetate	C109604	C_5_H_10_O_2_	102.1	718.7	711.7	215.672	1.15993	
36	E-2-Pentenal	C1576870	C_5_H_8_O	84.1	740.8	740.6	233.767	1.10517	
37	Methyl 2-methylbutanoate	C868575	C_6_H_12_O_2_	116.2	777.8	—	264.029	1.18837	
38	(Z)-3-Hexen-1-ol	C928961	C_6_H_12_O	100.2	842.0	—	336.689	1.23522	
39	(Z)-3-Hexen-1-ol	C928961	C_6_H_12_O	100.2	841.7	—	336.398	1.51614	
40	n-Hexanol	C111273	C_6_H_14_O	102.2	866.0	878.5	364.678	1.32262	
41	n-Hexanol	C111273	C_6_H_14_O	102.2	864.2	878.8	362.637	1.63724	
42	2,5-Dimethylpyrazine	C123320	C_6_H_8_N_2_	108.1	918.0	—	445.015	1.11588	
43	2-Heptenal (E)	C18829555	C_7_H_12_O	112.2	950.0	967	506.519	1.25442	
44	2-Heptenal (E)	C18829555	C_7_H_12_O	112.2	949.5	966	505.592	1.66892	
45	1-Octen-3-ol	C3391864	C_8_H_16_O	128.2	978.7	990	561.802	1.15748	
46	2-Pentylfuran	C3777693	C_9_H_14_O	138.2	988.7	—	581.012	1.25414	
47	2-Acetylthiazole	C24295032	C_5_H_5_NOS	127.2	1018.0	—	634.528	1.12647	
48	2-Octenal (E)	C2548870	C_8_H_14_O	126.2	1060.6	1061	710.704	1.33063	

“RI” represents the retention index calculated on FS-SE-54-CB column using n-ketones C4–C9 as external standard. “—” indicates that no reference value was found.

As shown in Tables 3, 4, these compounds represent six classes, including aldehydes, ketones, alcohols, esters, pyrazines, and other compounds. The generation of these volatile compounds was closely associated with the degradation of lipids and proteins through both enzymatic and non-enzymatic reactions (19). The volatile compound’s peak area of the DFSM was significantly higher than that of the AFSM, which might be because oil was added to the DFSM. Generally, oil could have a great influence on lipid oxidation, which is crucial to the generation of the typical aroma (33). Among them, the highest level of volatile compounds peak area appeared in the DF-190 (36714.12). The peak area of aldehydes in fried shrimp meat increased. The aldehydes peak area was found to increase remarkably as the frying temperature rose. It can be speculated that high temperatures could promote the generation of some aldehydes. DF-190 had the highest peak area for aldehydes (22201.41), which contains 2-methylbutanal, 3-methylbutanal, methylal, 2-octanal (E), benzaldhyde, and other substances. Esters were significantly lower in AFSM than in DFSM, and there was no significant effect of frying temperature on the peak area of esters. Alcohols was produced by the reaction of alkoxy radicals, generated during the process of lipid oxidation, with another fatty molecule (34). The alcohol peak area of the DFSM was the highest, followed by that of the CK group peak area (*p* < 0.05), and the AFSM was the lowest. In addition, the content of 2,5-dimethylpyrazine and 2-pentylfuran increased with the increase in temperature. These heterocyclic compounds might be produced by Strecker degradation and aldehyde condensation in the Maillard reaction during heating.

**FIGURE 5:**
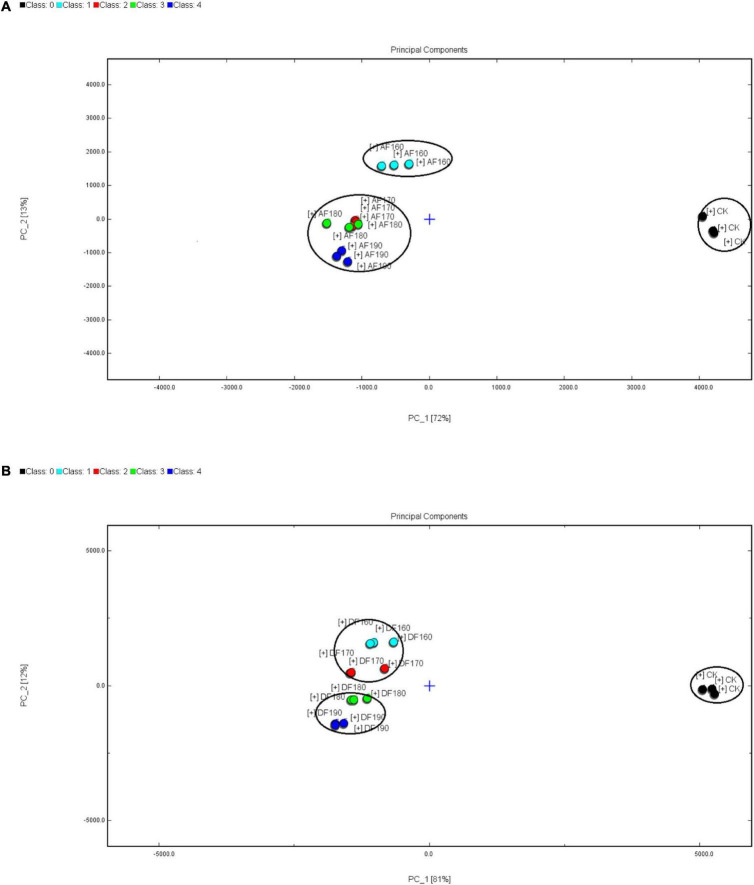
Effect of frying temperature on the principal component analysis (PCA) of AFSM **(A)** and the principal component analysis (PCA) of DFSM **(B)**. DF, shrimp meat was treated with deep frying; AF, shrimp meat was treated with air frying; 160, 170, 180, 190: The fried temperature of shrimp meat is expressed as 160, 170, 180, and 190°C.

The result of the PCA was shown in Figure 5, the volatile compounds show a difference among the frying shrimp meat. As shown in the figure, the volatile compounds of the AF and DFSM were quite significantly different from that of the CK group. In AF, the volatile compounds of shrimp meat fried at 160°C were significantly different from that fried at 170, 180, and 190°C. In DF, the volatile components of fried shrimp at 160 and 170°C were similar, and there was no significant difference between 180 and 190°C.

### Free amino acids analysis

Free amino acids are commonly used as quality indicators for various fish and crustacean species. The content of FAAs is essential for the evaluation of protein-rich products. The content of free amino acids in shrimp meat is shown in Table 5, and 16 FAAs were identified. The main FAAs in raw shrimp meat were arginine (1617.31 ± 3.47 mg/g) and histidine (80.00 ± 0.38 mg/g), which are associated with bitter taste (35). Six essential amino acids (EAAs) were included in the 16 FAAs, and the EAA content is the main factor affecting the nutritional value of the protein. In raw shrimp meat, the EAA content was 198.59 mg/g. During food processing, the composition and content of FAAs can change significantly depending on the processing conditions. The total content of FAAs in fried shrimp meat was obviously decreased compared to the control group (*P* < 0.05). These changes can be attributed to heating-induced protein denaturation, decomposition of FAAs, and different interactions between FAAs and the filling oil (36). Aubourg (37) reported that the filling medium and/or interaction with oxidized lipids may lead to the loss of FAAs. The total free amino acid (TFAA) content in AFSM was significantly higher than that in DFSM (*P* < 0.05), indicating that AF reduced the loss of TFAA compared with DF, which may be due to the interaction between FAAs and frying medium caused by DF treatment.

**TABLE 5:** Effect of frying method and temperature on the FAAs of shrimp meat.

Types	FAAs (mg/g)								
	
	CK	DF-160	DF-170	DF-180	DF-190	AF-160	AF-170	AF-180	AF-190
Aspartic Asp	1.87 ± 0.03^c^	0.60 ± 0.05^f^	3.13 ± 0.25^a^	1.18 ± 0.08^e^	0.55 ± 0.20^f^	1.57 ± 0.01^d^	2.62 ± 0.06^b^	1.67 ± 0.01^d^	1.62 ± 0.06^d^
Serine Ser	34.92 ± 0.42^a^	11.27 ± 0.18^g^	23.54 ± 1.47^d^	19.88 ± 0.49^e^	10.49 ± 2.57^g^	16.90 ± 0.12^f^	24.18 ± 0.18^d^	29.53 ± 0.11^b^	27.45 ± 0.24^c^
Glutamic Glu	18.69 ± 0.27^b^	10.50 ± 0.04^e^	22.72 ± 4.91^a^	11.05 ± 0.06^de^	6.32 ± 3.99^f^	18.23 ± 0.06^bc^	17.95 ± 0.12^bc^	14.64 ± 0.14^cd^	15.51 ± 0.09^bc^
Alanine Ala	69.60 ± 0.11^b^	10.64 ± 0.20^g^	130.09 ± 4.61^a^	31.38 ± 0.55^e^	16.24 ± 2.81^f^	38.87 ± 0.45^d^	48.39 ± 0.53^c^	36.80 ± 0.34^d^	47.00 ± 0.43^c^
Glycine Gly	72.26 ± 0.12^a^	14.06 ± 0.17^g^	64.43 ± 2.14^b^	26.09 ± 0.50^e^	18.62 ± 3.32^f^	39.26 ± 0.38^c^	39.59 ± 0.36^c^	34.49 ± 0.36^d^	38.59 ± 0.32^c^
Cysteine Cys	–	–	1.01 ± 0.08^a^	–	–	–	–	–	–
Valine Val	23.48 ± 0.02^a^	7.84 ± 1.60^e^	16.33 ± 0.36^c^	16.26 ± 0.11^c^	11.23 ± 1.07^d^	15.91 ± 1.50^c^	19.01 ± 1.37^b^	16.72 ± 1.35^c^	20.06 ± 1.37^b^
Methionine Met	45.47 ± 0.43^a^	9.82 ± 0.38^f^	22.68 ± 0.75^b^	13.08 ± 0.54^e^	13.20 ± 2.20^e^	20.58 ± 0.88^c^	16.28 ± 0.34^d^	22.44 ± 0.58^b^	20.13 ± 0.10^c^
Isoleucine Ile	15.77 ± 0.05^a^	1.84 ± 0.09^h^	8.37 ± 0.29^d^	7.26 ± 0.16^e^	4.81 ± 0.89^g^	6.59 ± 0.06^f^	11.49 ± 0.08^b^	7.04 ± 0.05^ef^	10.27 ± 0.09^c^
Leucine Leu	28.25 ± 0.08^a^	4.91 ± 0.08^g^	17.42 ± 0.53^c^	14.26 ± 0.38^d^	9.33 ± 1.54^f^	11.73 ± 0.06^e^	19.57 ± 0.23^b^	13.60 ± 0.11^d^	20.08 ± 0.17^b^
Tyrosine Tyr	50.14 ± 3.51^a^	17.35 ± 0.24^de^	26.96 ± 0.30^b^	10.42 ± 0.16^e^	18.73 ± 1.34^cd^	27.89 ± 0.07^b^	25.02 ± 11.02^bc^	11.37 ± 0.08^e^	12.49 ± 0.05^de^
Phenylalanine Phe	24.82 ± 0.68^a^	7.52 ± 0.34^i^	13.34 ± 0.34^e^	12.53 ± 0.35^f^	9.48 ± 1.04^h^	11.52 ± 0.11^g^	17.13 ± 0.14^c^	14.86 ± 0.02^d^	18.59 ± 0.16^b^
Lysine Lys	60.80 ± 0.02^a^	16.85 ± 0.30^g^	34.64 ± 1.18^b^	25.88 ± 0.46^e^	19.02 ± 3.50^f^	26.84 ± 0.25^e^	29.53 ± 0.27^d^	31.94 ± 0.29^c^	36.66 ± 0.30^b^
Histidine His	80.00 ± 0.38^a^	21.00 ± 0.43^h^	57.24 ± 1.56^b^	25.73 ± 0.64^g^	18.46 ± 3.16^i^	28.73 ± 0.23^f^	31.78 ± 0.24^e^	54.22 ± 0.48^c^	43.87 ± 0.33^d^
Arginine Arg	1617.31 ± 3.47^a^	410.08 ± 5.96^f^	1069.45 ± 35.45^b^	704.46 ± 12.80^e^	643.01 ± 121.40^e^	821.27 ± 8.19^d^	694.63 ± 6.83^e^	962.14 ± 9.29^c^	1048.39 ± 7.76^b^
Proline Pro	27.42 ± 0.16^b^	14.01 ± 0.19^e^	17.37 ± 0.35^d^	20.77 ± 0.68^c^	11.29 ± 5.07^e^	19.87 ± 0.19^cd^	25.48 ± 0.29^b^	30.94 ± 0.48^a^	30.71 ± 0.35^a^
TFAA	2170.8	558.29	1528.72	940.23	810.78	1195.76	1022.65	1282.4	1381.42
FAA	224.76	61.08	261.28	110.35	63.51	134.7	152.6	148.07	160.88
EAA	198.59	48.78	112.78	89.27	67.07	93.17	113.01	106.6	125.79

Values carrying different letters at the same time indicate statistically significant differences according to Duncan’s multiple range test (*p* < 0.05). DF, shrimp meat was treated with deep frying; AF, shrimp meat was treated with air frying; 160, 170, 180, 190: The fried temperature of shrimp meat is expressed as 160, 170, 180, and 190°C.

In fried shrimp meat, Ala, Gly, and Arg were the most abundant FAAs. Ala can be converted to acetaldehyde with a sweet taste by Strecker degradation (38). The acetaldehyde produced can further promote the formation of alkyl pyrazines with a strong aroma (39). DF-170 had the highest Ala content of 130.09 mg/g. Gly, together with hydroxyproline, is an indicator of the presence of connective tissue, principally collagen, and plays a key role in its stability (40). Gly is associated with a sweet taste (35). DF-170 had the highest Gly content of 64.43 mg/g. The amount of Arg in fried shrimp meat was significantly lower compared to the control group (*P* < 0.05). Shahidi found that Arg can be found in many types of seafood (41). Despite the bitter character, the presence of large amounts of Arg gives the food a palatable taste. When the concentration of some bitter amino acids is below their threshold, it can enhance the sweetness and taste of other amino acids, thus improving the overall taste (42). DF-170 and AF-190 had the highest Arg content of 1069.45 and 1048.39 mg/g, respectively.

It is noteworthy that Glu has a significant impact on flavor due to its low threshold (0.3) and synergistic effect with IMP. The loss of Glu is of great interest because although Glu is a non-essential amino acid, it is an important source of nitrogen that is associated with and even contributes to taste perception (43). Except for DF-170, AFSM had the lowest Glu loss. Cys can react with reducing sugars to produce furan, which can improve the overall flavor of fried shrimp meat. However, Cys was only detected in DF-170.

### Changes in sensory scores

**FIGURE 6:**
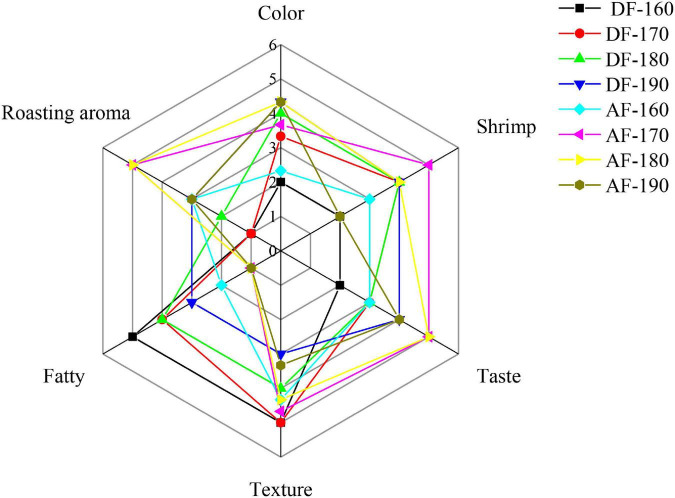
Effect of frying method and temperature on the sensory description of shrimp meat taste. DF, shrimp meat was treated with deep frying; AF, shrimp meat was treated with air frying; 160, 170, 180, 190: The fried temperature of shrimp meat is expressed as 160, 170, 180, and 190°C.

As shown in Table 6, it can be seen of overall acceptance that the sensory score for DFSM was close to AFSM, and the sensory scores reached the maximum when the frying temperature was 170°C, which were 4.18 and 4.20. In terms of color, both DF and AF frying methods scored between 3 and 4, with AF-180 and AF-190 scoring the highest, which is consistent with the results of b* values in color determination, indicating that yellowness value is one of the most important indicators for consumers when choosing fried products. The AFSM and DFSM were provided with a special roasting aroma and fatty aroma. The roasting aroma score of the AFSM was higher than that of the DFSM, the scores of AF-170 and AF-180 were the highest. But the fatty aroma score of the AFSM was lower than that of the DFSM, probably because volatile flavor contributed more in the DFSM, as analyzed by “GC-IMS.” However, at the lower frying temperature, DFSM was given a greasy feeling, which made the shrimp meat less sensual. The texture score of AFSM is significantly lower than that of DFSM. This is consistent with the results for surface hardness and elasticity, mainly due to the higher moisture loss in the AF treatment compared to the DF treatment, resulting in the formation of a hard outer shell with increased hardness and decreased elasticity of the samples. As can be seen from Figure 6, the texture property score decreases with increasing frying temperature under the same frying method, which is also due to higher moisture loss due to increasing temperature, thus making the hardness increase and elasticity decrease.

**TABLE 6:** Effect of frying method and temperature on sensory scores of shrimp meat.

Group	Sensory evaluation
CK	–
DF-160	3.31 ± 0.17^d^
DF-170	4.18 ± 0.00^a^
DF-180	4.01 ± 0.06^ab^
DF-190	3.95 ± 0.09^b^
AF-160	3.60 ± 0.00^c^
AF-170	4.20 ± 0.10^a^
AF-180	4.10 ± 0.17^ab^
AF-190	3.90 ± 0.20^b^

Values carrying different letters at the same time indicate statistically significant differences according to Duncan’s multiple range test (*p* < 0.05). DF, shrimp meat was treated with deep frying; AF, shrimp meat was treated with air frying; 160, 170, 180, 190: The fried temperature of shrimp meat is expressed as 160, 170, 180, and 190°C.

## Conclusion

The results showed that the evolution of the physicochemical and flavor characteristics of shrimp meat was significantly different in the temperatures of frying. Compared with DF, the AF gave shrimp meat the characteristics of low fat and high protein, and it could reduce the fat content by approximately 4.26–6.58 g/100g. The 16 FAAs were detected in fried shrimp meat. Except for DF-170, the total FAAs content of AFSM was significantly higher than that of DFSM shrimps (*p* < 0.05). The degree of lipid degradation in AF was lower in comparison with that in DF. But the texture characteristics of DFSM were better than AFSM. The 48 volatile components were detected in fried shrimp, and aldehydes, ketones, alcohols, and esters were the main contributors to the flavor of fried shrimp meat. The total amount of volatile compounds detected in DFSM was higher than that in AFSM. E-2-pentenal, 2-heptenal (E), and methyl 2-methylbutanoate were identified only in DFSM. The fat, protein content, and surface hardness of frying shrimp meat all increased with the increase in frying temperature. As judged by sensory evaluation, the shrimp meat AF at 170°C was the most popular among consumers. Overall, AF is a healthier frying method for fried foods and is a worthwhile alternative.

## Data availability statement

The original contributions presented in this study are included in the article/supplementary material, further inquiries can be directed to the corresponding authors.

## Author contributions

MZ: experiments, statistical analysis of data, and writing – original draft. GS: experiments, statistical analysis of data, and writing – original draft. YD: statistical analysis of data and writing – original draft. CW: experimental design and review and editing. YQ: experimental design, conceptualization, and visualization. GX: project proposal and research methodology. LW, WW, LS, and AD: review and editing. All authors contributed to the article and approved the submitted version.
